# Effectiveness of low vision rehabilitation on visual function and quality of life in Sub-Saharan Africa: a systematic review

**DOI:** 10.3389/fresc.2026.1878737

**Published:** 2026-08-26

**Authors:** Kwadwo Owusu Akuffo, Sylvia Agyekum, Sylvester Kyeremeh, Albert Kwadjo Amoah Andoh, Isaiah Osei Duah Junior, Josephine Ampomah Boateng, Nana Akwasi Owusu Mensah, Eldrick Adu Acquah, David Owiredu, Samson Darrah, Debora Abena Baidoo, Werner Eisenbarth

**Affiliations:** 1 Department of Optometry and Visual Science, College of Science, Kwame Nkrumah University of Science and Technology, Kumasi, Ghana; 2 Department of Psychology, College of Health and Human Sciences, North Carolina Agricultural and Technical State University, Greensboro, NC, United States; 3 University of Ghana Centre for Evidence Synthesis, Department of Epidemiology and Disease Control, School of Public Health, University of Ghana, Accra, Ghana; 4 Department of Applied Science and Mechatronics, HM Hochschule München University of Applied Sciences, Munich, Germany

**Keywords:** assistive devices, optical aids, quality of life, residual vision, vision rehabilitation programs, visual disability

## Abstract

**Aim:**

This systematic review evaluates the effectiveness of low vision rehabilitation on visual outcomes and quality of life in Sub-Saharan Africa (SSA).

**Data sources:**

Databases such as PubMed, Web of Science, CENTRAL, Google Scholar, and Scopus were searched from inception to May 2025. Searches were restricted to English-language publications.

**Review methods:**

Titles and abstracts were screened in Covidence, with data extraction performed independently by two reviewers and disagreements resolved by consensus. Methodological quality was assessed using Joanna Briggs Institute Critical Appraisal Tools for quasi-experimental and cross-sectional designs. Due to heterogeneity, findings were synthesized narratively following the SWiM (Synthesis Without Meta-analysis) framework.

**Results:**

From 48,601 records, only three studies met the inclusion criteria, underscoring a critical evidence gap. All reported improvements in visual outcomes with optical devices included enhanced distance and near acuity, faster reading speeds, and fewer reading errors. One study documented significant quality-of-life gains, particularly in social functioning, with an improvement of 76.6% on the National Eye Institute Visual Function Questionnaire-25 (NEI VFQ-25). Another study highlighted that many students previously enrolled in schools for the blind retained functional vision following intervention, reflecting both the efficacy of rehabilitation and the unmet need for accessible services.

**Conclusion:**

The available evidence suggests low vision rehabilitation with optical devices may improve visual function and social participation-related quality of life in SSA. Despite the scarcity of studies and limited evidence base, these preliminary findings highlight the potential value of rehabilitation services in advancing education, independence, and psychosocial well-being in the region.

**Systematic Review Registration:**

https://www.crd.york.ac.uk/PROSPERO/view/CRD42024496027.

## 1 Introduction

Visual impairment is an increasingly significant global public health challenge, with the number of affected individuals rising annually due to factors such as population aging and limited access to timely eye care (1, 2). Low vision (LV), as a specific type of visual impairment, is defined by the World Health Organization (WHO) as best corrected visual acuity in the better eye less than 6/18 but at least 3/60, and/or a visual field narrower than 20° (3). Individuals with LV retain usable sight and can benefit from interventions such as rehabilitation and assistive devices (4, 5). Although preventable or treatable through optical correction or surgery, uncorrected refractive errors and cataracts remain the leading causes of LV (6, 7). As of 2020, an estimated 20,442,000 people in Sub-Saharan Africa (SSA) were living with moderate to severe vision impairment (8). However, the majority lack access to affordable LV services and rehabilitation, underscoring a substantial unmet need for accessible LV services and rehabilitation strategies across the sub-region (9, 10).

LV imposes a substantial personal, social, and economic burden (11, 12). It significantly reduces quality of life (QoL) (13) and functional independence, increasing the risk of physical injuries such as falls (14), as well as psychosocial issues like depression and social isolation (5). Moreover, these effects, in turn, diminish productivity and life satisfaction (15). On a broader scale, residual vision contributes to major economic losses, with global productivity costs estimated at US$411 billion annually (16). In the absence of rehabilitation services, individuals with LV often rely on others for daily activities, further intensifying the strain on families and communities (17). Low vision rehabilitation (LVR) is a comprehensive, evidence-based intervention designed to enhance functional vision and improve quality of life among individuals with residual vision (13, 18). It is a problem-solving approach tailored to an individual's visual acuity, age, type of impairment, sociocultural background, and personal goals, with the aim of maximizing daily functioning despite declining vision (19, 20). It is typically delivered by a multidisciplinary team comprising optometrists, ophthalmologists, low vision specialists, occupational therapists, psychologists, assistive technology experts, vision rehabilitation therapists, and other allied health professionals (19). Beyond functional support, LVR addresses the emotional and psychosocial challenges of vision loss, promoting psychological adjustment, independence, and social participation (18, 21). Furthermore, such interventions can significantly enhance QoL by reducing dependency, improving mobility, and facilitating greater community engagement (22).

Despite its global recognition as an effective intervention, the implementation of LVR in SSA remains limited, underutilized, and insufficiently studied (23). Available research is sparse and largely consists of small, methodologically diverse studies lacking comprehensive evaluation (24). Previous reviews have examined low vision in SSA, focusing on service availability, accessibility (24) and barriers (23), while a separate review evaluated effectiveness of visual rehabilitation in low-and middle-income countries more broadly (25). However, no systematic synthesis has been conducted to assess the collective impact of these interventions in the SSA region. This review seeks to address that gap by critically examining the effectiveness of low vision rehabilitation in improving visual function and quality of life among individuals living with visual impairment in SSA.

## 2 Methods

This systematic review was conducted in accordance with the Preferred Reporting Items for Systematic Reviews and Meta-Analyses (PRISMA) checklist, ensuring transparency, methodological rigor, and reproducibility in the review process (26). Prior to data collection, the review protocol was prospectively registered with the International Prospective Register of Systematic Reviews (PROSPERO) under the registration number CRD42024496027. This registration outlines the objectives, eligibility criteria, data sources, and planned methods of analysis, thereby reinforcing the credibility and integrity of the review.

### 2.1 Types of studies

This review systematically included interventional studies conducted within SSA that evaluated the effectiveness of LVR compared to no intervention. Eligibility criteria have been defined according to Population (Participant), Intervention, Control and Outcomes (PICO) framework. Eligible study designs comprised randomized controlled trials (RCTs), quasi-experimental designs, and observational studies incorporating both pre- and post-intervention outcome assessments. Studies were excluded if they were narrative reviews, expert commentaries, conference abstracts lacking full text, or if they focused exclusively on medical or surgical interventions (e.g., cataract extraction, intraocular lens implantation) which fall outside the scope of rehabilitative care.

### 2.2 Participants

Studies were eligible for inclusion if they involved individuals with LV residing in any SSA country, irrespective of age or sex. LV was defined based on World Health Organization (WHO) criteria: best-corrected visual acuity worse than 6/18 but equal to or better than 3/60 in the better-seeing eye, and/or a visual field constricted to less than 20° from fixation (3).

### 2.3 Intervention

Eligible interventions comprised any structured LVR programs or services designed to optimize functional vision. These included optical aids such as handheld or stand magnifiers and telescopes; assistive technologies like portable and desktop video magnifiers; visual skills training; environmental adaptations; occupational therapy; psychological support or counselling; and comprehensive rehabilitation delivered by LV therapists in both clinic and home-based settings.

### 2.4 Control

Eligible studies incorporated comparators including no intervention, usual care without LVR, or baseline pre-intervention measures in single-group study designs.

### 2.5 Outcomes

The primary outcomes assessed in this review were vision-related quality of life (VRQOL), including but not limited to measures obtained using the National Eye Institute Visual Function Questionnaire-25 (NEI VFQ-25) instrument; broader health-related QoL encompassing functional and psychosocial well-being linked to vision loss; and objective measures of visual function improvement, including changes in visual acuity, contrast sensitivity, visual field, and performance on functional tasks such as reading speed and mobility. Secondary outcomes included user satisfaction with LVR services, where such data were reported.

### 2.6 Adverse events

No adverse events were documented in the included studies. Studies evaluating medical or surgical interventions, which carry inherent clinical risks, were excluded from this review.

### 2.7 Search methods

A systematic literature search was conducted across five electronic databases, PubMed, Web of Science, Cochrane CENTRAL, Google Scholar, and Scopus, covering all records up to August 2024, with an update in May 2025. Searches were restricted to English-language publications. The search strategy combined keywords including “low vision” OR “visual impairment,” “rehabilitation” OR “training,” and “Sub-Saharan Africa.” Detailed search terms and strategies are provided in the Supplementary Material (Appendix S1). Additionally, reference lists of included studies were manually screened to identify further eligible articles. Two independent reviewers searched and screened studies and any discrepancies between reviewers were resolved through a consensus or by a third reviewer.

### 2.8 Study selection and data collection

**Figure 1:**
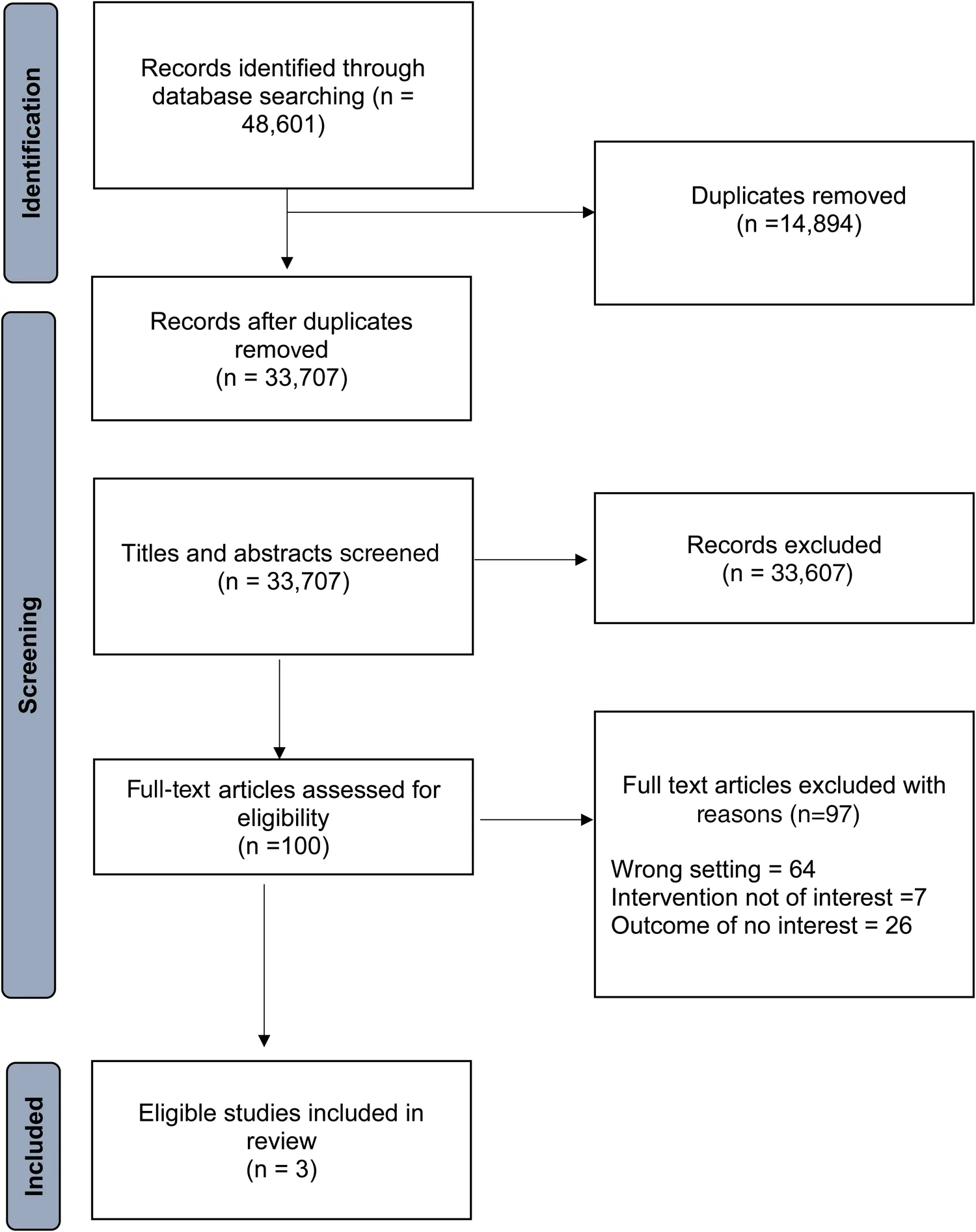
PRISMA flow diagram illustrating the process of study retrieval and selection.

All retrieved references were imported into Covidence, where duplicates were systematically removed. Titles and abstracts were independently screened by reviewers against predefined eligibility criteria, followed by a full-text assessment of potentially relevant articles. Data extraction was performed using a standardized form within Covidence, capturing key information on study design, participant characteristics, intervention details, outcomes measured, and reported results (Figure 1).

### 2.9 Data synthesis

Due to substantial heterogeneity in study designs, participant characteristics, intervention modalities, and outcome measures, a quantitative meta-analysis was deemed inappropriate, and a narrative synthesis was conducted based on SWiM guidelines (27). The included studies involved diverse age cohorts within varied clinical and educational contexts, utilizing heterogeneous outcome metrics, including objective clinical measures such as logMAR visual acuity and reading speed, alongside validated patient-reported outcome measures like NEI VFQ-25. Synthesis of findings was organized by key outcome domains, primarily visual function and VRQOL, with careful consideration of contextual factors and methodological variability to inform interpretation and generalizability.

### 2.10 Risk of bias assessment

The methodological quality of included studies was appraised using the Joanna Briggs Institute (JBI) Critical Appraisal Tools tailored for quasi-experimental and cross-sectional designs (28). The quasi-experimental checklist comprises nine criteria evaluating variable definition clarity, participant comparability, presence of control groups, consistency of usual care and outcome measurement, follow-up completeness, outcome measure reliability, and appropriateness of statistical methods. The cross-sectional tool includes eight items addressing clarity of inclusion criteria, detailed participant and setting descriptions, reliability of exposure assessment, identification and management of confounding factors, validity and reliability of outcome measures, and suitability of statistical analysis. Two independent reviewers assessed each study, rating items as ‘yes’, ‘no’, ‘unclear’, or ‘not applicable’, with discrepancies resolved by consensus discussion. A greater number of ‘yes’ ratings indicated higher methodological rigor.

## 3 Results

### 3.1 Study selection and characteristics

The electronic database searches initially retrieved 48,601 records. After removal of duplicates, 33,707 unique records remained, of which 33,607 were excluded based on title and abstract screening. Full-text review was conducted for 100 articles, resulting in the inclusion of 3 studies and exclusion of 97 studies due to reasons including irrelevant outcome measures (*n* = 26), non–SSA settings (*n* = 64), or interventions outside the scope of interest (*n* = 7). Ultimately, three studies met the inclusion criteria: one observational cross-sectional study, one quasi-experimental study, and one employing pre- and post-intervention design. Geographically, two studies were conducted in Ghana (29) and South Africa (30), respectively, while the third included participants from Kenya and Uganda (31) (Table 1). All studies evaluated optical low vision devices (LVDs) such as handheld magnifiers, stand magnifiers, and telescopes (hyperoculars), as part of the rehabilitation intervention. Only one study assessed vision-related quality of life (VRQOL) using the NEI VFQ-25 instrument. Reported outcomes included improvements in VRQOL and visual function measures. Participant ages ranged from 5 to 22 years. Across the included studies, LVR demonstrated significant positive effects on various functional and quality-of-life parameters.

**Table 1:** Characteristics of included studies.

Author, year	Country	Study design	Sample size	Type of intervention	Outcomes	Follow-up duration	Results
Silver et al. 1995 (31)	Kenya & Uganda	Cross-sectional study	140	Stand magnifiers, handheld	VFI	Not reported	Improved near visual acuity was recorded in approximately 36% of the participants; locally manufactured devices met two-thirds of need
Ovenseri-Ogbomo et al. 2016 (29)	Ghana	Pre and post-interview design	62 recruited; 25 obtained LVDs; 22 followed-up and assessed	Optical low vision devices^a^	VRQOL	3 months	There was a significant improvement in 8 of the 10 subscales investigated following low vision services
					VFI		Distance visual acuity improved from (0.60–1.68) LogMAR to (0.40–1.68) LogMAR
Urvashni Nirghin 2023 (30)	South Africa	Quasi-experimental design	15	Optical devices (hand- held and stand magnifiers)	VFI	Immediate assessment	Improved mean reading rate and reduced reading errors with low-vision optical devices

VFI, visual function improvement; VRQOL, vision-related quality of life.

a study did not specify the type of optical low vision device.

### 3.2 Quality of life

The impact of LVR on QoL was assessed in one study conducted in Ghana by Ovenseri-Ogbomo et al. (29). The study used a pre- and post-intervention design which evaluated changes in VRQOL following the provision of optical LVDs. A total of 62 individuals aged 14 years and older, all meeting the WHO criteria for low vision, were recruited between February 2011 and April 2012 through non-probability sampling. Among these, 25 participants (40.3%) received LVDs after low vision assessment, and 22 (88%) were available for follow-up three months later. While the specific types of devices were not described, all were optical aids prescribed as part of routine low vision care (29).

Outcomes were measured using the NEI VFQ-25, a validated instrument assessing VRQOL across multiple functional domains. Participants completed the questionnaire at baseline and three months post-intervention. The study found statistically significant improvements in 8 of the 10 NEI VFQ-25 subscales, including general vision, near and distance activities, peripheral vision, role difficulty, dependency, mental health, and social functioning (all *p* < 0.01). No significant changes were observed in color vision (*p* = 0.096).

The most substantial improvement was observed in the social functioning domain, which increased by 76.6% following intervention. Additionally, self-reported difficulty in vision-related tasks declined notably: the proportion of participants reporting extreme difficulty dropped from 59.1% to 9.1%, while those reporting little or no difficulty increased from 13.6% to 72.7% (*p* = 0.0001).

#### 3.2.1 Visual function improvement

In addition to VRQOL, Ovenseri-Ogbomo et al. (29) also evaluated objective improvements in visual function. Using logMAR charts, the study found a significant enhancement in distance visual acuity following the provision of optical LVDs. Mean (±SD) visual acuity improved from 1.04 (±0.26) logMAR at baseline to 0.83 (±0.28) logMAR post-intervention (*p* < 0.0001), corresponding to an average improvement of two lines on the logMAR scale.

A second study conducted by Nirghin et al. (30) in KwaZulu-Natal, South Africa, assessed the impact of optical devices on reading performance in school-aged children with LV. Using a quasi-experimental design, the study recruited children aged 15 to 19 years from two schools for the visually impaired. Following a comprehensive low vision evaluation, participants completed the English Paediatric Rate of Reading (PRR) test with and without optical devices. The assistive devices included hand-held and stand magnifiers, and basic user training was provided as needed. The mean (±SD) reading rate increased from 59.3 (±24.08) correct words per minute (cwpm) without a device to 67.1 (±25.6) cwpm with a device, while error rates decreased from 10 to 8 errors per minute. Although the reading rate improvement was not statistically significant (*p* = 0.087), the effect was clinically relevant, particularly given the strong correlation between device use and reading performance (r = 0.822, R^2^ = 0.675). The study also found that reading fluency improved progressively with grade level.

The third study, a cross-sectional investigation by Silver et al. (31), evaluated the unmet need for optical LVDs among students in schools for the blind in Kenya and Uganda. A total of 230 students aged 5 to 22 years were assessed, including 16 university-level participants. Of these, 140 students (61%) were classified as having low vision based on WHO criteria. Comprehensive low vision evaluations, including distance and near acuity testing, functional vision assessment, and refraction, revealed high levels of uncorrected refractive error: 58.6% had errors greater than 2 dioptres, and 25% had significant astigmatism. Refractive correction resulted in clinically meaningful acuity improvements in 59 students, though only 27 (19.3%) were considered to benefit substantially from new spectacles alone. Overall, 50 students (35.7%) were determined to require LVDs to read standard print. Near visual acuity testing demonstrated that 110 students (78.6%) could read N5–N8 print sizes when aided by refractive correction or LVDs, with 46 of these (41.8%) relying specifically on LVDs to attain this level of acuity. The devices prescribed included hand-held and stand magnifiers, high reading additions, and hyperoculars.

### 3.3 Risk of bias assessment of selected studies

The two quasi-experimental studies demonstrated moderate methodological quality (Table 2). Both established plausible cause-effect relationships, employed consistent and reliable outcome measures, and utilized appropriate statistical analyses. However, methodological limitations included the absence of control groups in both studies and incomplete follow-up in one, which may introduce potential biases and limit causal inference of identification or control for confounding variables, and insufficient detail in statistical analysis (See Table 2). In contrast, the cross-sectional study exhibited a higher risk of bias, primarily due to unclear inclusion criteria, lack of identification or control of confounding variables, and insufficient detail in the statistical analysis, as shown in Table 3.

**Table 2:** Joanna Briggs Institute Methodological Quality Appraisal for quasi-experimental studies.

S/N	JBI Critical Appraisal Checklist for quasi-experimental	Urvashni Nirghin (30)	Ovenseri-Ogbomo et al. (29)
1	Is it clear in the study what is the ‘cause’ and what is the ‘effect’ (i.e., there is no confusion about which variable comes first)?	Yes	Yes
2	Were the participants included in any comparisons similar?	Yes	Yes
3	Were the participants included in any comparisons receiving similar treatment/care, other than the exposure or intervention of interest?	Not applicable	Yes
4	Was there a control group?	No	No
5	Were there multiple measurements of the outcome both pre and post the intervention/exposure?	Yes	Yes
6	Was follow up complete and if not, were differences between groups in terms of their follow up adequately described and analyzed?	No	Yes
7	Were the outcomes of participants included in any comparisons measured in the same way?	Yes	Yes
8	Were outcomes measured in a reliable way?	Yes	Yes
9	Was appropriate statistical analysis used?	Yes	Yes

**Table 3:** Joana Briggs Institute Methodological Quality Appraisal for analytical cross-sectional studies.

S/N	JBI Critical Appraisal Checklist for analytical cross sectional studies	J. Silver et al. (31)
1	Were the criteria for inclusion in the sample clearly defined?	No
2	Were the study subjects and the setting described in detail?	Yes
3	Was the exposure measured in a valid and reliable way?	Yes
4	Were objective, standard criteria used for measurement of the condition?	Yes
5	Were confounding factors identified?	No
6	Were strategies to deal with confounding factors stated?	No
7	Were the outcomes measured in a valid and reliable way?	Yes
8	Was appropriate statistical analysis used?	No

Overall, the evidence is limited with observational studies, no RCTs and considerable methodological heterogeneity. As such, the findings of this review should be interpreted as preliminary and the certainty of evidence for the effectiveness of low vision rehabilitation in SSA remains low.

## 4 Discussion

This systematic review evaluated the effectiveness of LVR on visual function and QoL among individuals with low vision in SSA. A total of three studies met inclusion criteria, all of which focused on the use of optical LVDs as the primary rehabilitative intervention. Notably, none of the included studies evaluated non-optical rehabilitation approaches such as visual skills training, environmental modifications, assistive technologies, psychological support, or counselling, revealing a significant gap in the current evidence base.

Across all studies, the use of optical LVDs was consistently associated with improvement in visual function. Ovenseri-Ogbomo et al. (29) reported a statistically significant enhancement in distance visual acuity, with a mean improvement of two lines on the logMAR chart post intervention. Similarly, Nirghin et al. (30) documented a clinically meaningful increase in reading performance among school-aged children using optical aids, with reading rates rising by an average of 7.7 correct words per minute, though this improvement was not statistically significant. The increase in reading fluency suggests magnification may support educational development among school-aged children. In the study by Silver et al. (31), 78.6% of students with LV achieved functional near acuity (N5–N8), with 41.8% requiring LVDs to do so, demonstrating the critical role of assistive devices in enhancing access to printed materials. However, the study also highlighted underutilization of LV services in special education settings, where many students with usable residual vision were receiving Braille only instruction, thereby limiting opportunities for functional vision support (31).

QoL outcomes were assessed in only one study using the NEI VFQ-25. Ovenseri-Ogbomo et al. (29) observed significant improvements in eight out of ten subscales, particularly in domains related to general vision, social functioning, and mental health. The most substantial improvement was noted in social functioning (+76.6%), suggesting that LVR can meaningfully enhance psychosocial well-being and community participation (29). However, the ocular pain subscale did not show significant improvement (*p* = 0.348), indicating that some aspects of VRQOL may be less responsive to optical interventions alone.

These findings are consistent with global evidence. A study in India employing a multidisciplinary rehabilitation approach found significant improvements in visual functioning and QoL, as measured by the VA LV VFQ-48 and the IVI questionnaire. Although effect sizes were moderate (e.g., ES = 0.63 for overall IVI score), the results underscore the benefit of comprehensive rehabilitation models (32). Similarly, Lamoureux et al. (17) in Australia reported small-to-moderate improvements in domains such as reading and emotional well-being after a structured low vision program, with Cohen's d ranging from 0.17 to 0.30. However, no significant gains were observed in mobility and independence, potentially due to short follow-up durations or limited uptake of mobility training. In contrast, Ovenseri-Ogbomo et al. (29) demonstrated improvements in both social functioning and role difficulties, suggesting that optical-only interventions can produce broader psychosocial benefits in the African context.

A key difference between the studies reviewed in SSA and those conducted in high-income countries lies in the type, extent and multidimensionality of interventions (33). While multidisciplinary programs involving occupational therapy, orientation and mobility training, and assistive technology are commonly implemented in high-income and middle-income country settings (34, 35), the studies from SSA focused mainly on optical devices (24). This disparity likely reflects systemic differences in resource availability, service delivery infrastructure, and trained workforce capacity across the region (23). These systemic issues help contextualize the limited evidence identified in our review. We also consider the evidence from different settings as complementary, in that, studies from high income countries demonstrate what is possible with comprehensive resources whereas SSA studies demonstrate what is achievable with available and context-appropriate interventions. Nevertheless, the consistent improvements in visual acuity, reading ability, and quality of life suggest that even basic optical interventions can yield meaningful outcomes when appropriately deployed, particularly in resource-constrained environments (36–38). Notably, the absence of studies evaluating non-optical rehabilitation reflects a service gap in SSA and provides basis for further research to assess comprehensive rehabilitation approaches in the region.

Additional contextual evidence is provided by Yibekal et al. (39), who conducted a cross-sectional study among 484 Ethiopian adults with visual impairment. Although this study was not included in our review, as it did not evaluate intervention, it found that nearly half experienced poor VRQOL, particularly among rural residents and those with long standing vision loss. These findings highlight the substantial burden of LV and the urgent need for accessible rehabilitation services across underserved SSA populations.

Beyond clinical outcomes, the feasibility of LVD provision in SSA is influenced by local manufacturing capacity and the optical range of available devices, particularly when considering contextual feasibility and device appropriateness in resource-limited settings. Silver et al. (31) reported locally manufactured LVDs met the needs of approximately two-thirds of students with LV, yet 36% required devices with powers exceeding +28 dioptres, beyond what local production could offer. While low-cost LVDs developed by Christoffel Blindenmission (CBM) in Nairobi addressed many students’ needs, their limited optical range underscores the challenge of balancing cost, availability, and optical performance. Advanced manufacturing technologies needed to produce high-powered aspheric lenses remain largely inaccessible in many African settings, representing a significant barrier to scaling up effective LVD provision and consequently improving visual function and overall QoL.

### 4.1 Strengths and limitations

This review underscores the urgency of implementing scalable and sustainable low vision rehabilitation models across SSA, where services remain severely underdeveloped and fragmented. The findings should be considered preliminary as the overall certainty of evidence for the effectiveness of LVR in SSA remains low. Due to substantial heterogeneity in study designs, outcome measures, and intervention types, a meta-analysis was not feasible. A common limitation among the studies was the absence of control groups, which limits causal inference. The focus of studies on the school-aged population and the absence of studies on the adult population, who may have limited access to visual rehabilitation, is a noteworthy limitation. Additionally, small sample sizes, particularly in interventional studies, reduce statistical power and precision. The limited number of available studies points to a broader evidence gap, potentially reflecting both low rehabilitation service uptake and limited research output in the region. Furthermore, the review may be affected by publication bias as studies with positive findings are more likely to be published.

## 5 Conclusion

In conclusion, this review suggests that LVR, specifically through optical devices, contributes significantly to improvements in both visual function and quality of life for individuals with low vision in SSA. The results suggest evidence of the need to expand low vision services in the region, particularly by integrating comprehensive rehabilitation approaches into educational and healthcare systems. Scaling up access to appropriate and contextually feasible devices, alongside training and support services, holds promise for improving the lives of individuals with low vision in Africa and beyond. Future research should prioritize well-designed controlled studies (including longitudinal studies and RCTs), studies on adult populations, extended follow-up periods, and evaluation of non-optical interventions. These efforts would strengthen the evidence base and inform the development of scalable, context-appropriate rehabilitation models in the region.

## Data Availability

The original contributions presented in the study are included in the article/Supplementary Material, further inquiries can be directed to the corresponding authors.
