# Supplementary material for: Effectiveness of low vision rehabilitation on visual function and quality of life in Sub-Saharan Africa: a systematic review
Source: Front Rehabil Sci. 2026 Aug 26;7:1878737. doi: 10.3389/fresc.2026.1878737 (PMC13553853; doi:10.3389/fresc.2026.1878737)
Supplement: Supplementary file 1 [file Datasheet1.docx]

**Appendix 1: Search Strategies**

**Appendix 1.1. Pubmed, Web of Science, Scopus & CENTRAL**

1.	#1 Visual impairment or vision impairment or visual disability or low vision or vision disorder

2.	#2 Visually impaired persons

3.	#1 OR #2

4.	#4 Rehabilitation or low vision therap*

5.	#5 Aid or therap* or service or low vision aid or equipment or devices or training

6.	#4 OR #5

7.	#7 Africa or Sub-Sahara*

8.	#8 Nigeria or Ghana or Angola or Benin or Botswana or Burkina Faso or Burundi or Cameroon or Cape Verde or Central African Republic or Chad or Congo or Côte d'Ivoire or Equatorial Guinea or Eritrea or Ethiopia or Gabon or Gambia or Guinea or Guinea-Bissau or Kenya or Lesotho or Liberia or Madagascar or Malawi or Mali or South Africa or Sudan or Swaziland or Tanzania or Togo or Uganda or Zambia or Zimbabwe or Niger or Rwanda or Senegal

9.	#7 OR #8

10.	#3 AND #6 AND #9

**Appendix 1.2. Google Scholar**

1.	#1 Visual impairment or vision impairment or visual disability

2.	#2 low vision or vision disorder

3.	#3 Visually impaired persons

4.	#1 OR #2 OR #3

5.	#5 Rehabilitation or low vision therap*

6.	#6 Aid or therap*or service or low vision aid

7.	#7 Equipment or devices or training

8.	#5 OR #6 OR #7

9.	#9 Africa or Sub-Sahara*

10.	#10 Nigeria or Ghana or Angola or Benin or Botswana or Burkina Faso or Burundi or Cameroon or Cape Verde or Central African Republic or Chad or Congo or Côte d'Ivoire or Equatorial Guinea or Eritrea or Ethiopia or Gabon or Gambia or Guinea or Guinea-Bissau or Kenya or Lesotho or Liberia or Madagascar or Malawi or Mali or South Africa or Sudan or Swaziland or Tanzania or Togo or Uganda or Zambia or Zimbabwe or Niger or Rwanda or Senegal

11.	#9 OR #10

12.	#4 AND #8 AND #11

**Figure 1: PRISMA flow diagram illustrating the process of study retrieval and selection**.

Records identified through database searching (n = 48,601)

Duplicates removed

(n =14,894)

Records after duplicates removed

(n = 33,707)

Records excluded

(n = 33,607)

Titles and abstracts screened

(n = 33,707)

Full text articles assessed for eligibility

(n =100)

Full text articles excluded with reasons (n=97):

Wrong setting = 64

Intervention not of interest =7

Outcome of no interest = 26

Eligible studies included in review

(n = 3)

**Identification**

**Screening**

**Included**
